# Menopausal hormone therapy and breast cancer: what is the true size of the increased risk?

**DOI:** 10.1038/bjc.2016.231

**Published:** 2016-07-28

**Authors:** Michael E Jones, Minouk J Schoemaker, Lauren Wright, Emily McFadden, James Griffin, Dawn Thomas, Jane Hemming, Karen Wright, Alan Ashworth, Anthony J Swerdlow

**Affiliations:** 1 Division of Genetics and Epidemiology, The Institute of Cancer Research, London SW7 3RP, UK; 2 The National Cancer Registration Service–Eastern Office, Public Health England, Cambridge CB21 5XA, UK; 3 Division of Breast Cancer Research, The Institute of Cancer Research, London SW7 3RP, UK; 4 Breakthrough Breast Cancer Research Centre at The Institute of Cancer Research, London SW7 3RP, UK; 5 Division of Molecular Pathology, The Institute of Cancer Research, London SW7 3RP, UK

**Keywords:** bias (epidemiology), breast neoplasms, cohort studies, oestrogen replacement therapy

## Abstract

**Background:**

Menopausal hormone therapy (MHT) increases breast cancer risk; however, most cohort studies omit MHT use after enrolment and many infer menopausal age.

**Methods:**

We used information from serial questionnaires from the UK Generations Study cohort to estimate hazard ratios (HRs) for breast cancer among post-menopausal women with known menopausal age, and examined biases induced when not updating data on MHT use and including women with inferred menopausal age.

**Results:**

Among women recruited in 2003–2009, at 6 years of follow-up, 58 148 had reached menopause and 96% had completed a follow-up questionnaire. Among 39 183 women with known menopausal age, 775 developed breast cancer, and the HR in relation to current oestrogen plus progestogen MHT use (based on 52 current oestrogen plus progestogen MHT users in breast cancer cases) relative to those with no previous MHT use was 2.74 (95% confidence interval (CI): 2.05–3.65) for a median duration of 5.4 years of current use, reaching 3.27 (95% CI: 1.53–6.99) at 15+ years of use. The excess HR was underestimated by 53% if oestrogen plus progestogen MHT use was not updated after recruitment, 13% if women with uncertain menopausal age were included, and 59% if both applied. The HR for oestrogen-only MHT was not increased (HR=1.00; 95% CI: 0.66–1.54).

**Conclusions:**

Lack of updating MHT status through follow-up and inclusion of women with inferred menopausal age is likely to result in substantial underestimation of the excess relative risks for oestrogen plus progestogen MHT use in studies with long follow-up, limited updating of exposures, and changing or short durations of use.

Menopausal hormone therapy (MHT) provides effective relief from climacteric symptoms but some are associated with increased risk of stroke, venous thromboembolism, and breast, ovarian, and endometrial cancers (Medicines and Healthcare Products Regulatory Agency UK, 2007; Santen *et al*, 2010). The MHT prescribing decreased rapidly (Ameye *et al*, 2014) after its adverse effects on risk of breast cancer were highlighted in reports from the Women's Health Initiative trial (Rossouw *et al*, 2002) and the Million Women Study (Beral and Million Women Study Collaborators, 2003), but nevertheless it continues to be used by many women worldwide (Chlebowski and Anderson, 2012; Antoine *et al*, 2016).

Accurate information about the risks (and benefits) of MHT is essential to allow women to make informed decisions about their health, and evidence from observational studies is important because it is no longer justifiable to conduct long-term trials of MHT safety (Vickers *et al*, 2007). There are, however, shortfalls in the way information has been collected and analysed in most published epidemiological studies (Colditz *et al*, 1995; Magnusson *et al*, 1999; Li *et al*, 2000; Schairer *et al*, 2000; Daling *et al*, 2002; Newcomb *et al*, 2002; Beral and Million Women Study Collaborators, 2003; Li *et al*, 2003; Bakken *et al*, 2004, 2011; Chen *et al*, 2004, 2006; Stahlberg *et al*, 2004; Fournier *et al*, 2005; Brinton *et al*, 2008; Saxena *et al*, 2010; Beral *et al*, 2011; Calvocoressi *et al*, 2012; Ritte *et al*, 2012; Cordina-Duverger *et al*, 2013; Nyante *et al*, 2013; Fournier *et al*, 2014; Thorbjarnardottir *et al*, 2014; Roman *et al*, 2016). In prospective studies that only collect information on MHT up to the time of recruitment (e.g., cohort studies with no follow-up questionnaires) current use of MHT and duration of use may be misclassified because some never users of MHT will become users during follow-up, and some users will become ex-users; this may lead to biased assessment of breast cancer risk in relation to MHT use (Van Leeuwen and Rookus, 2003; Lee *et al*, 2005) but the extent has not been assessed empirically. In addition, analyses that include women who have had simple hysterectomy (i.e., without oophorectomy) before natural menopause will also lead to biased results (Collaborative Group on Hormonal Factors in Breast Cancer, 1997; Pike *et al*, 1998; Rockhill *et al*, 2000; Simpson *et al*, 2007) but many case–control and cohort studies include such women (Colditz *et al*, 1995; Magnusson *et al*, 1999; Li *et al*, 2000; Schairer *et al*, 2000; Daling *et al*, 2002; Newcomb *et al*, 2002; Beral and Million Women Study Collaborators, 2003; Li *et al*, 2003; Bakken *et al*, 2004, 2011; Chen *et al*, 2004, 2006; Stahlberg *et al*, 2004; Fournier *et al*, 2005; Brinton *et al*, 2008; Saxena *et al*, 2010; Beral *et al*, 2011; Calvocoressi *et al*, 2012; Ritte *et al*, 2012; Cordina-Duverger *et al*, 2013; Fournier *et al*, 2014; Thorbjarnardottir *et al*, 2014; Roman *et al*, 2016). Thus, although the epidemiological evidence clearly shows an increased risk of breast cancer with MHT use (Campagnoli *et al*, 2005; Greiser *et al*, 2005; Lee *et al*, 2005), there is uncertainty about the magnitude of the risk.

To address these issues we used serial questionnaire information from the Breakthrough Generations Study (BGS), which has ascertained MHT use and menopausal status at entry and during prospective follow-up, to estimate the relative risk of breast cancer in relation to MHT use among women whose age at menopause was known. We also assessed the likely extent of the biases that occur when only baseline questionnaire information is available and if women with simple hysterectomy are included in analyses.

## Materials and methods

The BGS is a cohort study of 113 693 women from the United Kingdom, aged ⩾16 years, from whom questionnaire information and informed consent was gained at recruitment during 2003–2015. The first follow-up questionnaire was completed at 2.5 years after recruitment, a second at ∼6 years, and a third at 9.5 years. The study was approved by the South East Multi-Centre Research Ethics Committee.

Breast and other cancers occurring in the cohort were identified from recruitment and follow-up questionnaires and spontaneous reports to the study centre. Confirmation of diagnosis was obtained from the cancer registries in the United Kingdom, ‘flagging' at the National Health Service Central Registers (virtually complete registers of the populations of England and Wales, and of Scotland, to which study participants can be linked and on which deaths and cancer registrations are ‘flagged' and then periodically reported to authorised medical researchers), pathology reports, and correspondence with patients' general practitioners.

Information on MHT was obtained at recruitment and from the second follow-up questionnaire, and was used to assess MHT exposure from menopause to date of breast cancer incidence or end of follow-up. Women were asked the ages or years they started and stopped episodes of MHT use, whether they were still using MHT, and the name of each drug used. We also asked participants at recruitment and in the second follow-up questionnaire how old they were when their periods stopped completely (i.e., they had gone 6 months without having had a period). Women who could not report age at natural menopause, because of hysterectomy without bilateral oophorectomy or because they were taking MHT or oral contraceptives around the time of menopause (i.e., at least 6 months without having a period and not being pregnant), were excluded from the main analyses because it was not possible to determine the age at which their ovarian function ceased. Information was also collected on other breast cancer risk factors (Swerdlow *et al*, 2011).

### Statistical analysis

The current analytic cohort is based on all women who were recruited to the study during June 2003 to December 2009 inclusive without previous breast cancer. The recruitment cutoff at December 2009 was selected because at the time of analysis the follow-up for the second questionnaire was practically complete for this group of recruits. Women with known age at menopause entered risk at their date of recruitment or menopause, whichever was later, and were censored at the earliest date of: first invasive or *in situ* breast cancer, death, follow-up questionnaire, or loss to study follow-up. Left-truncated and right-censored Cox proportional hazards regression (Cox, 1972) using attained age as the implicit timescale was used to estimate hazard ratios (HRs) and 95% confidence intervals (CIs) for MHT use and risk of first invasive or *in situ* breast cancer, adjusted for (continuous) menopausal age.

The MHT was analysed as a time-varying exposure lagged by 1 year (Schairer *et al*, 2000; Fournier *et al*, 2005). The effect of the lag was to make the year following start of treatment a nonexposed period (as the exposure within the early months of use is unlikely to be the cause of breast cancers occurring in that period), and the year after cessation to be considered a part of the period of exposure; this avoided a potential ‘reverse causation' bias, as MHT may be stopped during work-up to a formal breast cancer diagnosis. Follow-up time for each woman was divided into the period(s), after onset of menopause, that were: before MHT use, current MHT use, and after cessation of MHT use. A woman could move from being a user to ex-user to user again if there was a gap of at least 1 year between periods of MHT use (reports of MHT ending and re-starting within 1 year were treated as contiguous periods of use). In sensitivity analyses exploring the effect of not updating MHT use beyond baseline we assumed a duration of use as recorded at baseline and did not extrapolate the duration of use beyond that reported at baseline.

Drug preparation was assigned based on the type of MHT used longest during each contiguous episode of use. Tests for trend in duration of current use were calculated over periods of 0–<1, 1–<2,... years, scored as 0.5, 1.5,..., and included an indicator variable for current user *vs* never user (i.e., never users were not treated as users with zero duration but as a separate category to allow for systematic differences between those who are prescribed MHT and those who are not). Heterogeneity in HRs for subtypes of breast cancer by behaviour, oestrogen receptor status, and morphology was assessed using a data augmentation method (Lunn and McNeil, 1995). To assess potential confounding in addition to that due to age at menopause (as a continuous variable) we also adjusted for: birth cohort, benign breast disease, family history of breast cancer in first-degree relatives, socioeconomic score, age at menarche, age at first pregnancy and parity, duration of breastfeeding, oral contraceptive use before menopause, alcohol consumption, physical activity, pre-menopausal body mass index (BMI) at age 20 years, and post-menopausal BMI at recruitment (or if unavailable, from second follow-up questionnaire). We included any breast cancers diagnosed after recruitment (i.e., no minimum interval) but adjusted for, and tested for heterogeneity in, time since recruitment to BGS cohort (<1 year; 1+ year). We carried out additional analyses including women with uncertain menopausal age by including follow-up from these women only from age 58 years (the age by which 99% of women with known menopausal age were post-menopausal) but, to allow adjustment for menopausal age and estimation of duration of MHT use since menopause, we assumed menopause occurred at age 50 years (and only considered MHT use if it was used from this age). All statistical tests were two sided and analyses were conducted using Stata/IC version 14.0 (StataCorp, 2015).

## Results

During 2003–2009, a recruitment questionnaire was completed by 104 164 women who joined the BGS without prior breast cancer (or mastectomy), of whom 58 148 were established as post-menopausal before the censoring date for analysis. The first follow-up questionnaire at 2.5 years after recruitment was completed by 57 592 of these women (99.0%) and the second follow-up questionnaire at ∼6 years after recruitment was completed by 55 923 (96.2%) of these post-menopausal women (3.7% completed an abridged version by telephone). Of the remainder at second follow-up, 1.2% had died by this time, 1.7% were alive but had not completed the questionnaire although their vital and cancer status was available from ‘flagging' at the National Health Service Central Registers, and 0.9% were lost to follow-up at an earlier date (e.g., emigrations) or no follow-up was available. The follow-up rate (calculated as the total observed person-years divided by the person-years that would have been achievable if all post-menopausal women were followed-up to their second questionnaire or, if earlier, death) was 99.5%.

Menopausal age was known for 39 183 women and was uncertain (i.e., by hysterectomy, MHT, or oral contraceptive use) for 18 965 women. Of those with known menopausal age, 88.9% reported a natural menopause and 9.4% reported bilateral oophorectomy as the reason for menopause; the remaining 1.7% reported various other procedures or treatments. The median interval between menopause and subsequent recruitment for the 30 113 women known to be post-menopausal at recruitment was 7 years (interquartile range: 3 to 13 years). There were 9070 women pre-menopausal at recruitment who subsequently became post-menopausal during follow-up and at that point became eligible for analysis (median interval between recruitment and incident menopause for these 9070 women: 4 years; interquartile range: 2 to 5 years). The following results, unless otherwise stated, are based on the women with known menopausal age. The mean menopausal age was 50.2 (s.d.: 4.6) years and mean post-menopausal BMI was 25.7 (s.d.: 4.5) kg m^−2^. Table 1 presents further descriptive characteristics of the cohort and the pattern of MHT use at recruitment: 5.0% of women who had not used MHT by recruitment subsequently used MHT sometime during follow-up and 65.4% of current users at recruitment ceased use by end of follow-up. At recruitment the median duration of use of oestrogen-only MHT was 6.5 years (interquartile range: 2.5 to 10.5 years), of oestrogen plus progestogen MHT was 5.5 years (interquartile range: 2.5 to 9.5 years), and was 4.5.years (interquartile range: 1.5 to 8.5 years) for other types of MHT. Supplementary Table 1 describes the 775 first incident invasive or *in situ* breast cancers that were diagnosed during 204 390 person-years (median 6.0 years) of follow-up among 39 183 women with known age at menopause (and similar descriptive statistics for 18 965 women with unknown age at menopause).

The HR for invasive and *in situ* breast cancer adjusted for menopausal and attained age was 1.95 (95% CI: 1.55–2.46; *P*<0.001) for current users of all types of MHT relative to never users (Table 2). The HR increased with duration of current use by 3.8% per year (95% CI: 0.4–7.3% *P*=0.027) and at 15+ years was 2.02 (95% CI: 1.12–3.66; *P*=0.020). There was little difference in HR and trend after adjusting for further potential confounding variables (results shown in Supplementary Table 2; distribution of adjustment variables in Supplementary Tables 3 and 4), and hence the following results adjust only for menopausal and attained age. Risk was not significantly increased for past MHT use overall or by type of MHT preparation (Table 2), or oestrogen receptor status (Table 3), or morphological type (Supplementary Table 5), nor did the HR in past users differ by duration of past use (<5 years *vs* 5+ years: *P*_heterogeneity_=0.093).

Analysed by type of MHT preparation (Table 2), for oestrogen-only preparations (median duration of current use 6.6 years) breast cancer risk was not significantly increased with current use (HR=1.00; 95% CI: 0.66–1.54; *P*=0.99) and there was a nonsignificant increase in HR per year of use (4.2% per year of use; 95% CI: −1.8 to 10.5% *P*=0.17). For combined oestrogen plus progestogen preparations (median duration of current use 5.4 years) there was a significantly increased risk with current use compared with never use (HR=2.74; 95% CI: 2.05–3.65; *P*<0.001), and HR increased by 5.6% (95% CI: 1.2–10.2% *P=*0.011) per year of use, reaching HR=3.27 (95% CI: 1.53–6.99; *P*=0.002) at 15+ years of use. As can be seen by the non-overlapping CIs, there was a significant difference between the HRs for breast cancer risk in relation to current oestrogen-only MHT use *vs* never use compared with current oestrogen plus progestogen use *vs* never use (*P*_heterogeneity_<0.001). There was no heterogeneity by time since recruitment for risk of breast cancer in relation to oestrogen-only MHT use (*P*_heterogeneity_=0.35) or combined MHT use (*P*_heterogeneity_=0.72). Without adjustment for menopausal age, the HR for breast cancer and combined oestrogen plus progestogen MHT use was 2.64 (95% CI: 1.98–3.52; P<0.001).

The HR for combined oestrogen plus progestogen preparations was 2.96 (95% CI: 2.19–3.99; *P*<0.001) for invasive and 1.46 (95% CI: 0.53–4.00; *P*=0.47) for *in situ* breast cancer, but the difference between these was not significant (*P*_heterogeneity_=0.18) and *in situ* breast cancer only accounted for 14% of cases (Supplementary Table 6 presents results for invasive breast cancer only). The HRs for combined MHT for ductal (HR=2.60; 95% CI: 1.86–3.64; *P*<0.001) and lobular (HR=3.12; 95% CI: 1.50–6.51; *P*=0.002) breast cancers were both increased but not significantly different to each other (*P*_heterogeneity_=0.60; Supplementary Table 5). The HR for oestrogen receptor-positive breast cancer was 2.89 (95% CI: 2.09–4.00; *P*<0.001) and was not significantly different to that for oestrogen receptor-negative breast cancer (*P*_heterogeneity_=0.31; Table 3).

When analysed by type of combined oestrogen plus progestogen MHT, the HR for breast cancer for serial combined preparations (HR=2.95; 95% CI: 1.92–4.53; *P*<0.001) was not significantly different from that for continuous combined preparations (HR=2.67; 95% CI: 1.82–3.95; *P*<0.001) (*P*_heterogeneity_=0.74). For other or unspecified types of MHT, of which tibolone use made up 49%, risk compared with never users was significantly increased with current use (*P*<0.001), but there was no significant trend with duration of use (*P*=0.78). The HR for breast cancer for current tibolone use was 2.32 (95% CI: 1.03–5.20; *P*=0.041). Among women with no family history of breast cancer, the HR for current tibolone use was 1.60 (95% CI: 0.51–4.99; *P*=0.42) but this was based on only three cases of breast cancer, and among those with a positive family history of breast cancer the HR was 4.15 (95% CI: 1.32–13.1; *P*=0.015), but again was based on only three cases of breast cancer, and the difference between the HRs was not significant (*P*=0.25).

The above analyses update data on MHT use after recruitment using serial questionnaire information and exclude women with uncertain menopausal age (e.g., because of hysterectomy). We next examined the potential biases when these analytic procedures were not done (Table 4). The number of women available for analysis was reduced when using baseline information only because women who become post-menopausal after recruitment were not considered eligible, and the number of women available increased when those with uncertain menopausal age were included, although their follow-up for risk of breast cancer only started at age 58 years (only 13 404 women with uncertain menopausal age reached this age during follow-up; descriptive statistics in Supplementary Tables 3 and 4). The excess HR for breast cancer for combined MHT use was 53% smaller when usage during follow-up was ignored, 13% smaller when women with uncertain menopausal age were included in analyses, and 59% smaller for both these analytic approaches combined. If restricted to invasive breast cancer only, the biases were 46%, 15%, and 56% respectively. Table 4 also shows results by duration of use; at each duration there was ∼50% bias when usage during follow-up was ignored.

The interrelation between MHT, BMI, and risk of breast cancer is shown in Table 5. Risk of breast cancer increased significantly (*P*<0.001) with greater BMI among women not using MHT and among past users (*P*=0.030). The risk of breast cancer was increased with current use of combined oestrogen plus progestogen MHT within each category of BMI but there was no significant trend with BMI (*P*=0.39). The relative increase in risk for combined oestrogen plus progestogen MHT was smaller with greater BMI, for example, for BMI at +30kg m^−2^, the HR was (3.40/1.64=) 2.07 (95% CI: 0.84–5.10; *P*=0.11), BMI at 25 to <30 kg m^−2^ the HR was (3.49/1.36=) 2.56 (95% CI: 1.53–4.27), and for BMI <25 kg m^−2^ it was 3.27 (95% CI: 2.24–4.78; *P*<0.001), although the CIs overlapped and the trend with BMI was not significantly different to the trend among never users of MHT (*P*_heterogeneity_=0.79).

## Discussion

In our cohort, the risk of post-menopausal breast cancer was increased with current use of MHT, as in previous studies (Colditz *et al*, 1995; Collaborative Group on Hormonal Factors in Breast Cancer, 1997; Stahlberg *et al*, 2004; Campagnoli *et al*, 2005; Santen *et al*, 2010; Bakken *et al*, 2011; Beral *et al*, 2011; Salagame *et al*, 2011; Anderson *et al*, 2012; Chlebowski and Anderson, 2012), and the increase in risk was larger with combined oestrogen plus progestogen than with oestrogen-only formulations, again as seen before (Colditz *et al*, 1995; Olsson *et al*, 2003; Stahlberg *et al*, 2004; Campagnoli *et al*, 2005; Bakken *et al*, 2011; Beral *et al*, 2011; Chlebowski and Anderson, 2012; Roman *et al*, 2016). In particular, our relative risk estimates for current use of combined MHT are compatible with the results from epidemiological studies that have updated MHT use by questionnaire (Chen *et al*, 2004; Calle *et al*, 2009; Beral *et al*, 2011; Fournier *et al*, 2014) or record linkage (Roman *et al*, 2016). Relative risk increased with greater duration of current combined MHT use, to 3.27 at 15+ years of use, and these long-term risks were larger than those previously reported (Li *et al*, 2003; Brinton *et al*, 2008; Saxena *et al*, 2010), although there is considerable overlap in the CIs around these estimates.

We also found the association between risk of breast cancer and combined MHT use was compatible with previous publications that have shown attenuation of MHT-associated breast cancer risks in women with high BMI relative to non-users of MHT with similar BMI (Beral *et al*, 2011; Ritte *et al*, 2012); consequently, care is needed when comparing results between studies if they differ in BMI profile. Our results, however, should be comparable with other large epidemiological studies in this respect as the average post-menopausal BMI of women in our study (25.7 kg m^−2^) was similar to that in the Million Women Study (26.2 kg m^−2^; Beral *et al*, 2011), the European Prospective Investigation into Cancer and Nutrition (24.8 kg m^−2^; Ritte *et al*, 2012), and the Nurses' Health Study (24.9 kg m^−2^; Kotsopoulos *et al*, 2010), although notably lower than in the Women's Health Initiative trial (28.5 kg m^−2^; Rossouw *et al*, 2002). However, our results for women with BMI 25–<30 kg m^−2^ (HR=2.56; 95% CI: 1.53–4.27) can be compared with the Women's Health Initiative trial and observational study results for women who began combined MHT use within 5 years of menopause (HR=1.64; 95% CI: 1.00–2.68) (Prentice *et al*, 2008); our HR is larger but the CIs from both studies overlap.

We included invasive and *in situ* breast cancer as the analysis end point, similar to the Million Women Study (Beral *et al*, 2011), although when we re-analysed using only invasive breast cancer as the end point we saw modestly larger relative risks, but this did not materially change our conclusions (for direct comparability with studies that only include invasive breast cancer these results are available in Supplementary Table 6).

Previous studies have found greater risks for oestrogen receptor-positive than -negative breast cancer (Li *et al*, 2003; Chen *et al*, 2004; Fournier *et al*, 2008), and we also saw the same for combined MHT use, although the difference in risks in our study was not statistically significant perhaps because there were few current users of MHT who developed oestrogen receptor-negative breast cancer, and with 775 cases of breast cancer and only 52 cases currently using combined MHT the study lacked power to detect such statistical interactions. Our information on oestrogen receptor status also has some limitations because it came from cancer registration reports and the cutoffs used to define positive/negative were not standardised beyond that used in routine practice. We did not find overall increased risks stronger with combined continuous regimens than sequential ones, consistent with studies from the United Kingdom and the United States (Beral and Million Women Study Collaborators, 2003; Li *et al*, 2003; Campagnoli *et al*, 2005; Brinton *et al*, 2008; Opatrny *et al*, 2008), although studies from northern Europe have tended to show higher rates for continuous relative to sequential regimens (Olsson *et al*, 2003; Stahlberg *et al*, 2004; Campagnoli *et al*, 2005; Bakken *et al*, 2011; Roman *et al*, 2016) and the disparity may be because of differences in progestogen monthly dose (Bakken *et al*, 2004; Stahlberg *et al*, 2004; Campagnoli *et al*, 2005; Roman *et al*, 2016). The most frequently used serial regimens in our study were Prempak-C (conjugated oestrogen, 625 *μ*g and 1.25 mg; norgestrel, 150 *μ*g), Elleste Duet (estradiol, 1 and 2 mg; norethisterone, 1 mg), and Climagest (Estradiol, 2 mg; norethisterone, 1 mg), and the most frequently used continuous regimens were Premique (conjugated oestrogen, 625 *μ*g; medroxyprogesterone acetate, 5 mg), Kilovance (estradiol, 1 mg; norethisterone, 500 *μ*g), and Kilofem (estradiol, 2 mg; norethisterone, 1 mg).

We found significantly increased risk with other or unspecified types of MHT. There was, however, no significant trend with duration of use in this group, perhaps because the increased risk may be a characteristic of women who take these other types of preparation rather than an effect of the preparation itself. The most common type of MHT in this group was tibolone, a selective tissue oestrogenic activity regulator, and evidence suggests that in the United Kingdom it is preferentially prescribed to women at increased risk of breast cancer (Velthuis-Te Wierik *et al*, 2007).

Our MHT exposure information was gained at recruitment and from follow-up questionnaire 6 years later. As 96% of women completed this follow-up it seems unlikely that misclassification of exposure in the remaining 4% would have materially influenced our results. Some of our MHT usage was collected after diagnosis of breast cancer and this has potential to introduce recall bias. If women with breast cancer were more motivated to recall past exposures we may expect to have seen increased risks for oestrogen-only MHT *and* combined MHT separately but the HR for oestrogen-only MHT use was not increased. However, given the attention that the combined MHT has received in the lay press, women with breast cancer may preferentially recall combined MHT use. However, we note that only 10 out of the 52 women diagnosed with breast cancer, and who were current users of combined MHT, first started MHT use after recruitment to the study and the other 42 reported current use at recruitment, before their breast cancer diagnosis. It therefore seems unlikely that our results are appreciably affected by recall bias. Follow-up for vital and breast cancer status was obtained for 99% of participants and confirmation of reported breast cancers for 98%, and hence there was little scope for biases from unascertained mortality or exits, or erroneous reporting of breast cancer. A large proportion of women were excluded from our main analysis because their age at menopause was uncertain or unknown, usually because they started MHT or had hysterectomy without oophorectomy, before cessation of menstrual bleeding. A benefit gained by their exclusion is that our analysis was not diluted by the inclusion of possibly misclassified pre-menopausal women. Furthermore, the exclusion of women with unknown age at menopause does not produce biased results in prospective studies because these women could legitimately not have been recruited to a cohort if they were deemed ineligible by protocol; however, as a consequence our results may be less generalisable to all post-menopausal women. This lack of generalisability also affects other studies that have considered it inappropriate to include these women in their main analyses of MHT and breast cancer risk (Collaborative Group on Hormonal Factors in Breast Cancer, 1997; Pike *et al*, 1998; Ross *et al*, 2000; Rosenberg *et al*, 2006).

There are potential biases in cohort studies that only collect information on history of MHT use up to the time of recruitment (e.g., with no follow-up exposure data) that can seriously misclassify actual use (Van Leeuwen and Rookus, 2003; Lee *et al*, 2005). Magnitude of effect in randomised controlled trials may also suffer from bias because of misclassification of exposure if adherence to treatment is not complete. For example, in the Women's Health Initiative trial of combined MHT, 42% of women in the active treatment arm discontinued MHT, and 10.7% of women in the placebo arm started MHT, at some point during the active intervention (Rossouw *et al*, 2002).

By using information from follow-up questionnaires we were able to update MHT status and duration right up to the breast cancer diagnosis or censoring date, and hence avoid the misclassification that would occur if only baseline questionnaire information was available. Without such follow-up information (i.e., as in many published cohorts (Beral and Million Women Study Collaborators, 2003; Bakken *et al*, 2004, 2011; Stahlberg *et al*, 2004; Brinton *et al*, 2008; Saxena *et al*, 2010; Nyante *et al*, 2013)) we found that the excess breast cancer risk for combined MHT would be underestimated by ∼53%. The Women's Health Initiative randomised clinical trial saw a similar degree of underestimation in risk of breast cancer for oestrogen plus progestogen treatment relative to placebo when comparing an analysis making allowance for nonadherence to MHT with an intention-to-treat analysis (49% increased risk of breast cancer *vs* a 24% increased risk; i.e., 51% reduction in excess relative risk) (Chlebowski and Anderson, 2012).

Biases may also occur if analyses included women with simple hysterectomy before menopause (i.e., without oophorectomy) or who started MHT before cessation of menstrual bleeding (Collaborative Group on Hormonal Factors in Breast Cancer, 1997; Pike *et al*, 1998; Rockhill *et al*, 2000; Simpson *et al*, 2007), as most published cohort and case–control studies have done (Colditz *et al*, 1995; Magnusson *et al*, 1999; Li *et al*, 2000; Schairer *et al*, 2000; Daling *et al*, 2002; Newcomb *et al*, 2002; Beral and Million Women Study Collaborators, 2003; Li *et al*, 2003; Bakken *et al*, 2004, 2011; Chen *et al*, 2004, 2006; Stahlberg *et al*, 2004; Fournier *et al*, 2005; Brinton *et al*, 2008; Saxena *et al*, 2010; Beral *et al*, 2011; Calvocoressi *et al*, 2012; Ritte *et al*, 2012; Cordina-Duverger *et al*, 2013; Fournier *et al*, 2014; Thorbjarnardottir *et al*, 2014; Roman *et al*, 2016). We found that the consequent underestimation of the excess HR for combined MHT use, because of not adjusting adequately for menopausal age, was ∼13%. However, women with simple hysterectomy, or those who started MHT before cessation of menstrual bleeding, may be different to women with known age at menopause, and this could be responsible for some or all of the bias.

For the combination of both the above types of bias we found that excess breast cancer risks would be underestimated by 59%. However, the uncertainty around this estimate is large (we estimate 35–83%), and furthermore study-specific characteristics (e.g., when study was conducted, duration of follow-up, etc.) are also likely to affect the size of bias that might be seen in other studies. The misclassification of current use of MHT may be less important in older studies conducted before results were published from the Women's Health Initiative trial (Rossouw *et al*, 2002) and Million Women Study (Beral and Million Women Study Collaborators, 2003) as following these reports women stopped using MHT in large numbers (Chlebowski and Anderson, 2012; Ameye *et al*, 2014). However, current advice suggests women should use MHT for the shortest possible time (Stuenkel *et al*, 2015), and thus observational studies conducted post 2002/2003 should be cautious about assuming MHT use at baseline will continue for the duration of the study.

A number of cohort studies have reported relative risks for breast cancer of ∼2.0 to 2.5 for ⩾10 years of combined MHT use (Beral and Million Women Study Collaborators, 2003; Brinton *et al*, 2008; Santen *et al*, 2010; Saxena *et al*, 2010; Bakken *et al*, 2011), but this is presumably an underestimate because all of these studies did not update MHT use through follow-up and included in analyses women with uncertain menopausal age. If correction is made for the bias this induces, we estimate the relative risk would be in the range 2.7 to 3.5, similar to the 3.5 we observed for 10+ years of use. However, at shorter durations of use too we saw appreciable bias and underestimation of the relative risk for combined MHT use.

In conclusion, our results show that risk of breast cancer increases with duration of use of combined MHT up to ⩾15 years, and relative risks in most of the published literature are likely to be substantially underestimated because of lack of updating MHT status through follow-up in cohort studies and inclusion of women with inferred menopausal age in cohort or case–control analyses. These results provide further information to allow women to make informed decisions about the potential risks and benefits of MHT use.

## Figures and Tables

**Table 1:** Characteristics of women from the Breakthrough Generations Study who were recruited during 2003–2009 and became post-menopausal before end of follow-up

	**Women with known age at menopause**		**Women with unknown age at menopause**	
**Study population**	***N***	***%***	***N***	***%***
**Year of birth**				
1908–1939	4303	11.0	2111	11.1
1940–1949	14 626	37.3	7922	41.8
1950–1959	16 692	42.6	6775	35.7
1960–1985	3562	9.1	2157	11.4
**Year of entry to analytic follow-up^a^**				
2003–2004	890	2.3	13	0.1
2005–2006	17 060	43.5	910	4.8
2007–2008	12 567	32.1	3101	16.4
2009–2015	8666	22.1	9380	49.5
Not included in analytic follow-up	0	0.0	5561	29.3
**Age at start of analytic follow-up (years)^a^**				
22–44	890	2.3	0	0.0
45–54	14 430	36.8	0	0.0
55–64	18 033	46.0	7824	41.3
65–74	5051	12.9	4939	26.0
75–98	779	2.0	641	3.4
Not included in analytic follow-up	0	0.0	5561	29.3
**Family history of breast cancer in first-degree relative**				
None reported	32 614	83.2	16 019	84.5
Yes	6569	16.8	2946	15.5
**History of benign breast disease**				
None reported	29 455	75.2	13 752	72.5
Yes	9728	24.8	5213	27.5
**Age at menarche (years)**				
7–11	8091	20.7	4177	22.0
12–14	23 665	60.4	11 122	58.6
15–19	3806	9.7	1784	9.4
Not known	3621	9.2	1882	9.9
**Parity**				
Nulliparous	5404	13.8	2110	11.1
Parous	33 724	86.1	16 825	88.7
Not known	55	0.1	30	0.2
**Oral contraceptive use**				
None reported	7576	19.3	3385	17.9
Previous use	31 607	80.7	15 580	82.2
**Post-menopausal MHT use at recruitment^b^**				
No previous use	20 114	66.8	5414	36.2
Ex-user	5771	19.2	5495	36.7
Current user: oestrogen only	1719	5.7	1905	12.7
Current user: oestrogen plus progestogen	1612	5.4	1491	10.0
Current user: other or unspecified MHT	398	1.3	211	1.4
Dates used unknown	499	1.7	440	2.9
**Total number of subjects**				
	39 183	100.0	18 965	100.0

Abbreviation: MHT=menopausal hormone therapy.

a Start of analytic follow-up based on latest of recruitment date or date became post-menopausal, or for women with unknown age at menopause, date became aged 58 years.

b Excludes women pre-menopausal at recruitment (9070 among those with known menopausal age; 4009 among those with unknown menopausal age).

**Table 2:** Relative risk^a^ of (invasive and *in situ*) post-menopausal breast cancer, by type of MHT preparation, in relation to MHT duration and recency of use, in 39 183 women from the Breakthrough Generations Study who were recruited during 2003–2009

	**Type of MHT preparation**											
	**Oestrogen only**			**Oestrogen plus progestogen**			**Other and unknown MHT**			**All types of MHT**		
**MHT use**	**No. of cases**	**HR**	**95% CI**	**No. of cases**	**HR**	**95% CI**	**No. of cases**	**HR**	**95% CI**	**No. of cases**	**HR**	**95% CI**
No previous use	Baseline group: no previous use^b^									500	1.00	Baseline
Currently using	23	1.00	0.66–1.54	52	2.74	2.05–3.65	15	3.04	1.81–5.33	90	1.95	1.55–2.46
Currently using MHT: by duration of current use (years)^c^												
>0–4	7	0.80	0.38–1.69	14	1.71	1.00–2.92	8	2.96	1.47–5.97	29	1.50	1.03–2.19
5–9	6	0.96	0.43–2.16	19	3.53	2.23–5.60				29	2.29	1.57–3.34
10–14	6	1.41	0.62–3.17	12	3.70	2.07–6.04	7	3.12	1.48–6.60	20	2.49	1.57–3.92
15+	4	1.14	0.42–3.08	7	3.27	1.53–6.99				12	2.02	1.12–3.66
Increase in HR per year of use among current users (trend)^d^												
		4.2%	−1.8–10.5%		5.6%	1.2–10.2%		*1.3%*	−7.6–11.1%		3.8%	0.4–7.3%
Past use	44	0.96	0.70–1.31	99	1.02	0.82–1.28	29	0.81	0.55–1.18	172	0.97	0.81–1.16
Past use of MHT: by time since last use (years)^e^												
1	2	0.40	0.10–1.62	10	1.61	0.86–3.01	4	0.42	0.16–1.11	12	0.92	0.52–1.63
2–4	18	1.02	0.63–1.63	30	1.09	0.76–1.59				52	0.99	0.74–1.32
5–9	17	0.99	0.61–1.62	33	0.80	0.56–1.15	14	1.06	0.62–1.82	64	0.89	0.68–1.17
10+	7	1.35	0.63–2.86	26	1.30	0.86–1.95	11	0.88	0.48–1.62	44	1.17	0.84–1.62
Increase in HR per year since last use among past users (trend)^f^												
		2.1%	−6.7–11.8%		−1.0%	−6.9–5.2%		1.4%	−6.8–10.3%			
Dates of use unknown	0			2	1.95	0.49–7.83	11	1.26	0.69–2.30	13	1.23	0.71–2.13

Abbreviations: CI=confidence interval; HR=hazard ratio, adjusted for attained age and age at menopause; MHT=menopausal hormone therapy.

a Hazard ratios estimated using Cox proportional hazards regression with attained age as timescale.

b Baseline group is ‘No previous use' as in analysis for ‘All types of MHT'.

c Duration of MHT current use was analysed as a time-varying exposure lagged by 1 year.

d Trend and intercept fitted for current users relative to hazard in those when no previous use (person-time for past use and missing dates when MHT used are excluded from trend analysis).

e The period up to the first year after cessation is considered to be part of the period of current use because exposure (current use) is lagged by 1 year.

f Trend and intercept fitted for past users relative to hazard in those with no previous use (person-time for current use and missing dates when MHT used are excluded from trend analysis).

**Table 3:** Relative risk^a^ of (invasive and *in situ*) post-menopausal breast cancer, by oestrogen receptor status, in relation to MHT, in 39 183 women from the Breakthrough Generations Study who were recruited during 2003–2009

	**Breast cancer oestrogen receptor status**								
	**Oestrogen receptor positive**			**Oestrogen receptor negative**			**Status unknown**		
**MHT use**	**No. of cases**	**HR**	**95% CI**	**No. of cases**	**HR**	**95% CI**	**No. of cases**	**HR**	**95% CI**
No previous use	374	1.00	Baseline	71	1.00	Baseline	55	1.00	Baseline
Currently using oestrogen only MHT	20	1.19	0.75–1.88	2	0.49	0.12–2.03	1	0.50	0.07–3.66
Currently using oestrogen plus progestogen MHT	41	2.89	2.09–4.00	5	1.70	0.68–4.24	6	3.19	1.36–7.44
Currently using other and unknown MHT	12	3.30	1.85–5.87	3	3.83	1.20–12.28	0		
Past use	128	0.94	0.76–1.16	27	1.01	0.63–1.62	17	1.11	0.63–1.97
Dates of use unknown	9	1.12	0.58–2.18	3	1.86	0.58–5.93	1	0.91	0.13–6.62

Abbreviations: CI=confidence interval; HR=hazard ratio, adjusted for attained age and age at menopause; MHT=menopausal hormone therapy.

a Hazard ratios estimated using Cox proportional hazards regression with attained age as timescale.

**Table 4:** Relative risk^a^ **of (invasive and *in situ*) post-menopausal breast cancer, by duration of current use of oestrogen plus progestogen MHT, for analyses with and without MHT updated through follow-up and inclusion or exclusion of women with uncertain menopausal age, in women from the Breakthrough Generations Study who were recruited during 2003–2009**

	**MHT use updated through follow-up, women with uncertain menopausal age excluded**			**No updating of MHT use beyond baseline, women with uncertain menopausal age excluded**				**MHT use updated through follow-up, women with uncertain menopausal age included**				**No updating of MHT use beyond baseline, women with uncertain menopausal age included**			
	**(Number of women=39 183)**			**(Number of women=39 183)**				**(Number of women=52 587)**^b^				**(Number of women=43 484)**^b^^,^^c^			
**MHT use**	**No. of cases**	**HR**	**95% CI**	**No. of cases**	**HR**	**95% CI**	**Bias**^d^	**No. of cases**	**HR**	**95% CI**	**Bias**^d^	**No. of cases**	**HR**	**95% CI**	**Bias**^d^
No previous use	500	1.00	Baseline	525	1.00	Baseline		584	1.00	Baseline		531	1.00	Baseline	
Currently using	52	2.74	2.05–3.65	62	1.82	1.39–2.38	53%	73	2.51	1.96–3.21	13%	101	1.71	1.38–2.13	59%
Currently using oestrogen plus progestogen MHT: by duration of current use (years)^e^															
1–4	14	1.71	1.00–2.92	22	1.32	0.86–2.02	56%	15	1.62	0.97–2.71	13%	30	1.26	0.87–1.82	63%
5–9	19	3.53	2.23–5.60	25	2.35	1.56–3.53	47%	26	2.91	1.95–4.33	25%	35	1.70	1.20–2.40	72%
10+	19	3.54	2.21–5.65	15	2.34	1.38–3.97	47%	32	2.97	2.07–4.28	22%	36	2.51	1.77–3.55	40%

Abbreviations: CI=confidence interval; HR=hazard ratio, adjusted for attained age and age at menopause; MHT=menopausal hormone therapy.

a Hazard ratios estimated using Cox proportional hazards regression with attained age as timescale.

b Women with uncertain age at menopause only contributed follow-up from age 58 years.

c Women who become post-menopausal after recruitment were not considered eligible for this analysis.

d Bias in excess relative risk: ((HR0-1)-(HR1-1))/(HR0-1) where HR0 is the HR estimated when MHT use is updated through follow-up and women with uncertain menopausal age excluded, and HR1 is the biased HR.

e Duration of MHT use was lagged by 1 year in all analyses, and analysed as a time-varying exposure only when MHT use was updated through follow-up

**Table 5:** Relative risk^a^ **of (invasive and *in situ*) post-menopausal breast cancer, in relation to MHT, by post-menopausal BMI in 39 183** b **women in the Breakthrough Generations Study who were recruited during 2003–2009**

	**Post-menopausal BMI (kg m**^−**2**^)									**Trend**			
	**<25**			**25–<30**			**30+**			**(HR per unit increase in BMI)**			
**MHT use**	**No. of cases**	**HR**	**95% CI**	**No. of cases**	**HR**	**95% CI**	**No. of cases**	**HR**	**95% CI**	**HR**	**95% CI**	***P*****-value (trend)**	***P*****-value (heterogeneity)**^c^
No previous use	203	1.00	Baseline	188	1.36	1.12–1.66	100	1.64	1.29–2.08	1.04	1.02–1.06	<0.001	—
Currently using oestrogen only MHT	16	1.52	0.91–2.54	1	0.17	0.02–1.21	5	2.25	0.92–5.52	0.97	0.90–1.05	0.51	0.17
Currently using oestrogen plus progestogen MHT	31	3.27	2.24–4.78	16	3.49	2.10–5.82	5	3.40	1.40–8.26	1.03	0.96–1.12	0.39	0.79
Currently using other and unknown MHT	10	4.46	2.36–8.43	5	3.84	1.58–9.32	0			0.98	0.83–1.15	0.78	0.41
Past use	88	1.18	0.91–1.53	44	0.85	0.61–1.19	38	1.94	1.37–2.76	1.04	1.00–1.07	0.030	0.91
Dates of use unknown	7	1.50	0.71–3.19	6	2.04	0.91–4.61	0			0.93	0.80–1.09	0.39	0.18

Abbreviations: BMI=body mass index; CI=confidence interval; HR=hazard ratio, adjusted for attained age and age at menopause; MHT=menopausal hormone therapy.

a Hazard ratios estimated using Cox proportional hazards regression with attained age as timescale.

b A total of 1041 women (2.7%) were excluded from analysis because BMI was not known.

c Trend compared with the trend for no previous use.
